# Exploring the utility of different bulking agents for speeding up the composting process of household kitchen waste

**DOI:** 10.1038/s41598-025-85433-0

**Published:** 2025-01-20

**Authors:** Sania Mussa, Muhammad Farhan, Shoaib Ahmad, Khadija Zahra, Amina Kanwal, Qaiser Farid Khan, Muhammad Afzaal, Abdul Wahid, Pallab K. Sarker, Mohamed A. El-Sheikh, Shafaqat Ali

**Affiliations:** 1 Sustainable Development Study Center, Government College University Lahore, Lahore, Pakistan; 2 State Key Laboratory of Pollution Control and Resource Reuse, School of the Environment, Nanjing University, Nanjing, 210023 Jiangsu China; 3 Department of Botany, Government College Women University Sialkot, Punjab, Pakistan; 4 Department of Microbiology, Ikam ul Haq Institute of Industrial Biotechnology, Government College University, Lahore, Pakistan; 5 Department of Environmental Science, Bahu din Zakaria University, Multan, Pakistan; 6 Environmental Studies Department, University of California Santa Cruz, Santa Cruz, CA USA; 7 Botany and Microbiology Department, College of Science, King Saud University, Riyadh 11451, Saudi Arabia; 8 Department of Environmental Sciences, Government College University Faisalabad, Faisalabad, 38000 Pakistan; 9 Department of Biological Sciences and Technology, China Medical University, Taichung, 40402 Taiwan

**Keywords:** Kitchen waste, Aerobic composting, Feedstock size, Bulking agents, Maturity, Ecology, Environmental sciences

## Abstract

Household kitchen waste (HKW) is produced in large quantity and its management is difficult due to high moisture content and complex organic matter. Aerobic composting of HKW is an easy, efficient, cost-effective and eco-friendly method. This study is designed to achieve a zero-waste concept and to convert HKW. We optimized the type and size of three different bulking agents to speed up the composting process. The tested bulking agents were fallen leaves, sawdust and fly ash. The results showed a higher and longer thermophilic phase (55^o^C) for 11 days in C2. Higher moisture content (69%) and higher organic matter degradation (38.4%) were also observed in C2. The pH range in all compost treatments was 7-8.5, Electrical conductivity range was 1.8–3.55 mS/cm, C/N ratio range was 15.4–18.1, water holding capacity range was 3.25–4.3 g water/g dry sample, total potassium range was 1.52–1.61%, total phosphorous range was 0.83–1.14%. The highest germination index (119.1%) was also obtained in C2. The highest chili height (16.7 cm), greater number of leaves (20), greater shoot fresh weight (4.75 g) and root fresh weight (1.2 g) was obtained in the presence of C2. Similarly, greater water WHC (2.8 g water/g DW), higher porosity (55.49%) and higher aggregate stability (54.14%) of soil was also obtained by C2. This research effectively reduced the maturation time to 32 days and converted kitchen waste into compost (resource). This is a very practical idea for home composting and kitchen gardening to combat food security issues in developing countries.

## Introduction

Household Kitchen Waste (HKW) is generated in food processing, food making, consumption and edible remains from homes. HKW consists of vegetable or fruit peels, spoiled fruit or vegetables, egg shells, bones etc. Due to the increasing population, rapid social development and food-making techniques the HKW has increased globally^1^. According to the Food and Agricultural Organization, one billion three hundred million tons of HKW is generated every year globally^2^. HKW is a major challenge to treat or manage due to huge amounts of water (59.2-82.7%), carbonaceous substances (around 51% carbon), lignin compounds, viruses and bacteria^3^. Most of the HKW is dumped openly without any treatment and causing severe environmental pollution^4^. This starts deteriorating easily and cause problems such as leaching, malodor, release of unpleasant gases, growth of viruses, mosquitoes, potential spread of pathogenic bacteria and epidemic^5^. Therefore, an efficient method to manage HKW is the need of the hour. The concept of waste to resource is gaining popularity and is sustainable way of waste management. There are different traditional methods of treating kitchen waste such as open dumping, landfill, burning, incineration, anaerobic digestion^6^. These methods cause serious environmental problems and are unproductive for recycling resource. Therefore, these methods are exchanged by methods that are more efficient, sustainable and environmentally friendly^7^. Among these methods, most consistent method is aerobic composting of HKW. Aerobic composting is a natural process that degrade waste through biological processes under control conditions and converts organic material in to valuable compost, in presence of air^3^. HKW composting is a proper and suitable method to manage waste^8^.

Kitchen waste has unfavourable physical/chemical properties such as huge amount of water, low carbon nitrogen ratio, low porosity, compact assembly, so it is not composted well alone^9^. To overcome these problems bulking agents are used. Bulking agents (BA) are amendments in composting that control the physio-chemical properties of HKW to enhance composting process^10^. Bulking agents generate inter-particle spaces, create air space in waste material, balance carbon and nitrogen ratio and control the moisture content of waste^11^. There is range of different BA that can be used in kitchen waste composting to speed up the composting process. These bulking agents include rice husk, paper, coffee grounds, tea waste, straw, bean dregs^12^, sawdust (SD)^13^, grain hull^14^, minerals, fly ash, green waste (mowed grass, grass clippings, fallen leaves, tree pruning, branch cuttings, weeds)^15,16^. Use of fly ash is well reported in literature, like coal fly ash^17,18^ and biomass fly ash^19,20^. Fly ash reduces the bio-availability of heavy metals^17^, reduces the lignin in compost better than control^19^, assist the reshaping and proliferation of microbial communities^18^, neutralizes the acidic soil and reduce the number of exchangeable aluminum and hydrogen ions^20^. If waste (of any type) is not well managed it caused environmental pollution and life of all forms is disturbed. Therefore, if we use these different types of waste in a process that lessons its impacts and produce a valuable product then it will be better than throwing the waste without any treatment^10^. Type and size of feedstock is a significant factor for efficient composting. Smaller size of waste leads to large surface area, uniformity, proper aeration and increase moisture content that makes it easier for microbes to degrade efficiently. The size of waste material can be reduced by cutting, chopping, crushing, shredding etc^10^.

Home composting is an effective method to treat small volume of HKW under control conditions at source^6^. Composting of HKW at home can also reduce the emission of CH_4_ and N_2_O during degradation^21^, this depends on adopted scheme and method of composting^22^. The main objectives of this study were to 1). optimization of kitchen waste composting at household level 2). investigating the effectiveness of different bulking agents (fallen leaves, fly ash, saw dust) on composting process 3). Explore usefulness of synthesize composts on soil properties and plant growth.

## Materials and methods

### Raw materials

**Fig. 1:**
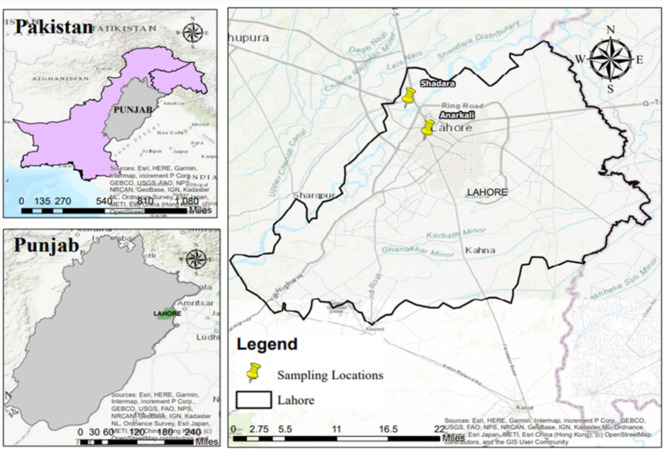
Study site and selected sampling points.

The HKW used in this study was collected from household in Shahdara and from Anarkali food street Lahore (Fig. 1). Before processing, non-degradable materials (plastics, glasses) were removed from waste. The HKW used in this study contains egg shells, vegetable and fruit peels. Fallen leaves were collected from university garden. Saw dust was taken from a local carpenter’s workshop. Fly ash was taken from a local cafe^23^.

### Composting process and sampling

The half of HKW was cut into small pieces (5–10 mm) and another half was crushed (1–3 mm). The aerobic indoor composting experiment was conducted at the Environmental Microbiology Lab of Sustainable Development Study Center, Government College University Lahore. The composters were mud pots of 30 × 30 cm (length x diameter) having removable lid with a hole on it for aeration. Six experimental treatments were setup in triplicates naming C1, C2, C3, C4, C5 and C6. In each treatment HKW and bulking agents were mixed in ration of 70:30 by weight (Table 1). The pile of compost in each treatment was then mixed thoroughly on regular intervals of time to avoid anaerobic conditions. The moisture content was maintained by adding the required amount of water. Samples from each treatment was taken from three levels (surface, mid and base) and were then mixed to make composite sample. Samples were collected in transparent zipper bag and stored at 4^o^C for further testing^24^.

**Table 1:** Compost treatments and their composition.

Treatments	Composition				Replicates
	Kitchen waste size	Kitchen waste volume	Bulking Agent	Bulking Agent volume	
C1	5–10 mm	70%	Fallen Leaves	30%	C1R1, C1R2, C1R3
C2	1–3 mm	70%	Fallen Leaves	30%	C2R1, C2R2, C2R3
C3	5–10 mm	70%	Saw dust	30%	C3R1, C3R2, C3R3
C4	1–3 mm	70%	Saw dust	30%	C4R1, C4R2, C4R3
C5	5–10 mm	70%	Fly ash	30%	C5R1, C5R2, C5R3
C6	1–3 mm	70%	Fly ash	30%	C6R1, C6R2, C6R3
C (Control)	1–3 mm	100%	---	---	CR1, CR2, CR3

### Physical and chemical parameters analysis

Temperature of compost was measured by thermometer. pH and EC were measured by a multimeter in 1:10 (w/v) water-soluble extract^25^. Total nitrogen was measured using kjeldahl method^7^. Moisture content was calculated by Eq. 1^25^. Organic matter was quantified using loss on ignition (LOI) method at 550^o^C in muffle furnace (Eq. 2)^26^ ash content and total carbon were measured by using Eqs. 3 and 4, respectively^7^.

1 $$\:Moisture\:content=\frac{w-d}{w}$$

Where;

w = wet weight.

d = dry weight.

2 $$\:Organic\:matter\:\left(\%\right)=\:100-ash\%/$$

Ash % was determined by Eq. 3.

3 $$\:Ash\:\left(\%\right)=\:\frac{W3-W1}{W2-W1}\:X\:100$$

Where;

W1 = weight of clean dry crucible.

W2 = oven dry weight of sample and dry crucible.

W3 = ash weight (weight of ash and crucible).

4 $$\:Organic\:carbon\:\left(\%\right)=organic\:matter\:value\:X\:0.50$$

To determine potassium in compost sample, 0.5 g of compost was taken in a beaker and 4 ml nitric acid HNO_3_ was added in it and digested on hot plate at 145 C for 2 h. After digestion, filtered it through Whatman filter paper-42 and raised its volume to 50 ml by adding distilled water. Then potassium was analyzed by flame photometer.

Water Holding Capacity was calculated using Eq. 5^27^.

5 $$\:Water\:holding\:capacity=\:\frac{[\left(Ws-Wi\right)+MC\:X\:Wi]}{\left[\left(1-MC\right)X\:Wi\right]}$$

6 $$\text {WHC} \text{= [(Ws -Wi)+MC} \times \text{Wi/[(1-MC)}\times \text{Wi]}$$

Where;

Wi = initial wt. of compost.

Ws = final wt. of compost.

MC = Moisture content.

Germination index was determined using Eq. 7^28^.

7 $$\:\text{G}\text{e}\text{r}\text{m}\text{i}\text{n}\text{a}\text{t}\text{i}\text{o}\text{n}\:\text{i}\text{n}\text{d}\text{e}\text{x}\:\left(\text{G}\text{I}\right)=\frac{\sum\:\text{G}\text{s}}{\text{D}}$$

Where.

Gs = total number of germinated seeds.

D = number of days.

### Germination experiment

A pot experiment was set up to analyze the comparative impacts of composts on soil and plant growth. Six treatments were arranged as T1, T2, T3, T4, T5, T6. Soil was amended with compost C1, C2, C3, C4, C5, C6 in T1, T2, T3, T4, T5 and T6, respectively. Soil with no compost was used as a control (T0). Mixture of compost and garden soil was filled in pots (30 × 30 length x width) Five seeds of green chili were sowed in each treatment, all treatments were conducted in replicates. Moisture was controlled by spraying the required amount of water^12^. The germination experiment lasts for 40 days. Height of plants was measured by using meter rod from base to leaf of plant. Number of leaves were counted on each plant. To measure fresh weight of root and shoot the plants were harvested, washed with distilled water to remove soil. Then plants were dried in air for some time to remove water on their surface. Later, the root and shoot were separated and both were weighed separately. All the readings were taken in triplicates^6^.

### Soil analysis

Soil analysis was analyzed after plant harvesting. The soil was taken from each treatment and following soil analysis were measured:

Water holding capacity (WHC) was determined by the Eq. 5^27^. Porosity of soil was determined by taking a graduated cylinder of 100 ml and fill it about half with soil sample. Then tap the cylinder with fingers many times so that soil become settle in cylinder. Then volume (V) of the packed sample is note down. Then pour out the soil sample and save it. Then fill the cylinder with water up to 70 ml. Then slowly add the saved sample into the cylinder. Stir it with rod to break the clumps. Then leave it stand to allow bubbles to escape for five minutes. Note the final “volume of soil sample / water mixture”.

The porosity of the soil was calculated by Eq. 8.

8 $$\text {Porosity of sample\,}\%=\text{V of pore space (ml)}/\text{V of packed sample (ml)}\times100$$

$$\text {V of pore space (ml) \,}=\text{V of packed sample}-\text{V of solids}$$

$$\text {V of solids (ml) \,}=\text{V of soil sample}/\text{water mixture}-\text{70 ml of water}$$

The wet sieving technique was used to determine aggregate stability of soil^29^. Soil was sieved 8–12 mm size. Then 70 g of soil was taken and soaked on 2 mm sieve and raised and lowered for 2 min. Then 2 mm size aggregates were soaked on 250 μm sieve for 2 min. Capture the two aggregate fractions through filtration and dry them and weigh them. Aggregate stability was determined by Eq. 9.

9 $$\text {Soil aggregate stability \%\,}=\%\,\text{in 2mm fractions}+\%\,\text{in 250}\mu\text{m fractions}$$

$$\%\,\text {in 2 mm fractions}=\text{(wt. of fraction obtained on 2 mm sieve)}/\text{(70 g}-\text{stones wt)}\times 100$$

$$\%\,\text {in 250}\,\mu\text{m fractions}=\text{(wt. of fraction obtained on 250}\mu\text{m sieve)}/\text{(70 g}-\text{stones wt)}\times 100$$

### Statistical analysis

All the data is the average and standard deviations of three replicates. Data was processed and analyzed by using Microsoft Excel 2016 and SPSS version 16. ANOVA, Post Hoc test, Tuckey Test, LSD and descriptive analysis were performed^23^.

## Results and discussion

### Changes in the physio-chemical properties of compost

**Fig. 2:**
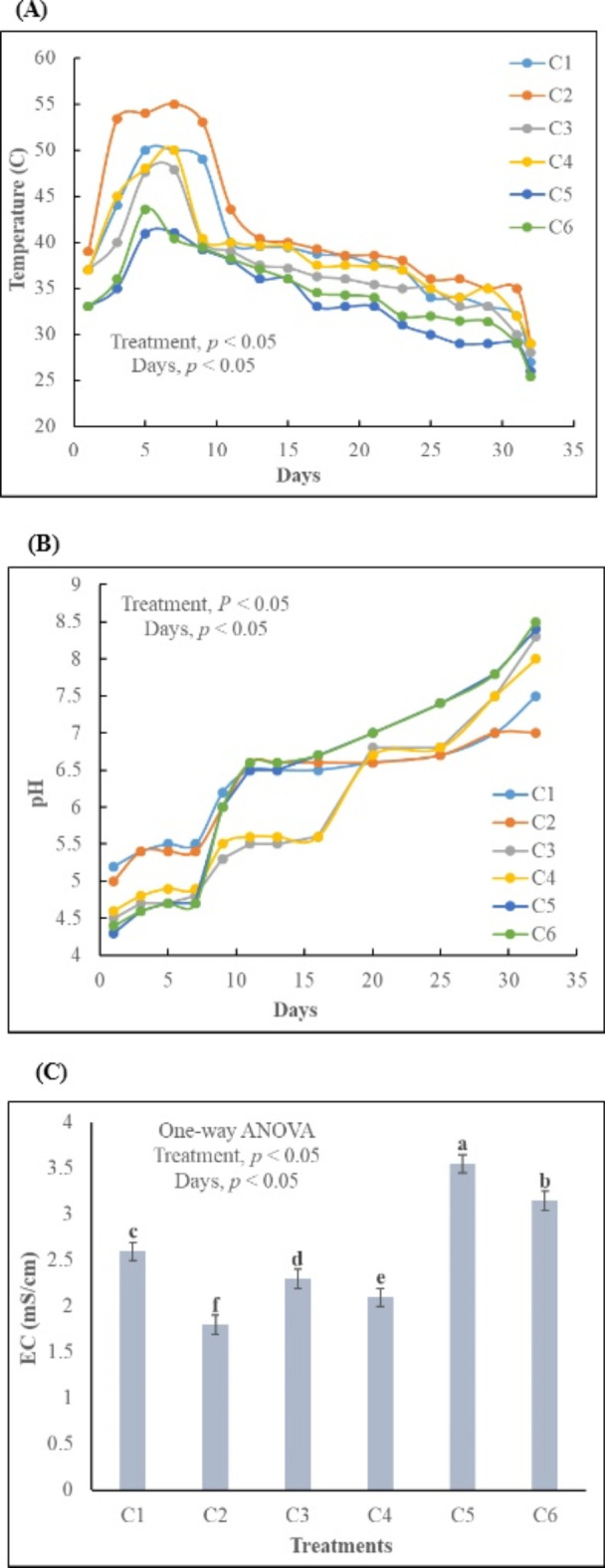
Changes in different parameters during the composting process, **(A)** temperature changes, **(B)** pH changes, and **(C)** electrical conductivity. C1 = kitchen waste + fallen leaves (5–10 mm), C2 = Kitchen waste + fallen leaves (1–3 mm), C3 = Kitchen waste + saw dust (5–10 mm), C4 = Kitchen waste + saw dust (1–3 mm), C5 = Kitchen waste + fly ash (5–10 mm), C6 = Kitchen waste + fly ash (1–3 mm). All the values are the mean of triplicate. Error bar represents the Standard Deviation. The alphabet above bars represents the Duncan multiple range test.

The temperature experienced significant variation across different treatments (*p* < 0.05). C2 exhibited a notably abrupt temperature rise compared to other treatments, while C4 underwent a sharp transition from the thermophilic phase to the cooling phase. In contrast, C5 displayed a more gradual temperature change than the other treatments. Only C2 maintained a consistently high temperature (50–60 °C) for over a week (Fig. 2A). The abrupt temperature changes in C3 and C4 may be attributed to the presence of saw dust, known for its high water absorption potential, causing rapid temperature fluctuations^5^. The elevated temperature results from microbial activities^30^, facilitated by the high surface area of fallen leaves and saw dust^31^. Control (lacking a bulking agent), failed to achieve a prolonged thermophilic phase necessary for pathogen elimination^30^. Fallen leaves and sawdust are rich in organic matter (OM) which enhances microbial and enzymatic activity^32^, and results in rise of temperature^33^. pH fluctuation was reported between 4.5 and 8.5 during composting process due to various reactions in the compost pile^34^. pH values were significantly different in all treatments (*p* < 0.05). C1 gradually shifted from acidic to alkaline pH, while C6 exhibited abrupt pH changes. In C4, pH turned acidic in the mesophilic phase, gradually becoming alkaline (Fig. 2B). C2 maintained an acidic pH for an extended period, possibly due to low mineralization^7^, high organic acid concentration^32^, slow degradation of organic N and low OM degradation rate^35^. C5 and C6 experienced a pH shift towards alkaline as the thermophilic stage approached. This change may be due to high temperatures, aerobic conditions, organic nitrogen mineralization and organic matter degradation^14^. Electrical conductivity (EC) of mature compost reflects its salt content and is crucial for explaining its impact on plant growth^6^. EC is directly related to pH values, with the increase in pH the EC also rises^35^. The high salinity > 4 mS/cm is not good for plants, the EC of final compost should be < 4 mS/cm^10^. EC values for C2, C3 and C4 remained within a safe range of 0-2.5 mS/cm, while C5, having flyash, had an EC value exceeding 3.5 mS/cm (Fig. 2C). High EC values pose a risk of soil salinity, have potential to harm or even kill the plants^28^. The higher EC range in C5 may be because the fly ash releases significant amounts of soluble ions^36^ and can accumulate different ions due to its extensive surface area^30^.

### Changes in moisture content (MC) and water holding capacity (WHC) during composting

**Fig. 3:**
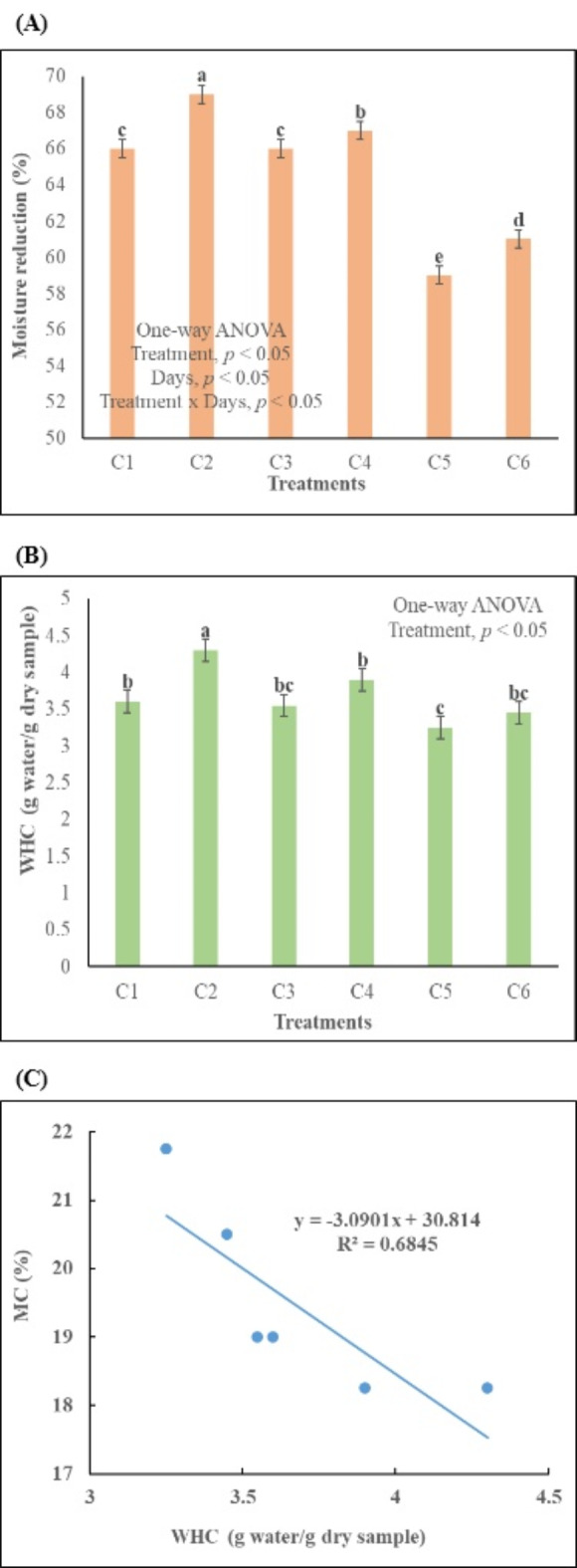
Changes in moisture content and water holding capacity during composting process, **(A)** moisture content reduction in different treatments, **(B)** water holding capacity in different treatments, **(C)** correlation between moisture content and water holding capacity. C1 = kitchen waste + fallen leaves (5–10 mm), C2 = Kitchen waste + fallen leaves (1–3 mm), C3 = Kitchen waste + saw dust (5–10 mm), C4 = Kitchen waste + saw dust (1–3 mm), C5 = Kitchen waste + fly ash (5–10 mm), C6 = Kitchen waste + fly ash (1–3 mm). All the values are the mean of triplicate. Error bar represents the Standard Deviation. The alphabet above bars represents the duncan multiple range test.

The moisture content plays a pivotal role in determining both the success of composting and the nutrient composition of the resulting compost. Elevated moisture content leads to nutrient leaching and hinders the attainment of higher temperatures. Across all treatments, the moisture content remained within the optimal range, except for C5 and C6 (Fig. 3A and C; Table 2). It is crucial for the moisture content to fall within the 50–65% range at the beginning of the composting process to prevent nutrient loss through leaching^37^. Towards the end of composting, the moisture content should ideally be in the 15–25% range, a criterion not met by C6. Moisture content indirectly influences the water holding capacity (WHC) and porosity^25^ of the final compost (Fig. 3). As moisture content increases, both porosity and WHC tend to decrease^38^. WHC varied significantly among all treatments (*p* < 0.05), with C2 exhibiting the highest water holding capacity. A high WHC is desirable to minimize nutrient loss, enhance aggregate size, and improve soil structure^5^. In this study, the WHC of all treatments differed significantly from each other (*p* < 0.05), with C5 having a very low WHC, while C2 displayed the highest WHC. This discrepancy is attributed to the superior surface area and adsorption capacity of bulking agents^39^. Another crucial parameter which influences soil quality and plant growth is porosity and it is dependent on both moisture content^25^ and bulk densities^27^. Optimal porosity and WHC conditions are directly proportional to the type and quantity of the bulking agent^38^.

**Table 2:** Changes in soil properties with the addition of different compost treatments.

Treatments	Water Holding Capacity (g water/g dry sample)	Soil Porosity (%)	Aggregate Stability of soil (%)
C1	2.2 ± 0.24c	54.35 ± 0.35b	52.32 ± 0.23b
C2	2.8 ± 0.43a	55.49 ± 0.28a	54.14 ± 0.43a
C3	2.1 ± 0.27 cd	53.8 ± 0.58c	52.11 ± 0.65de
C4	2.4 ± 0.71b	54.37 ± 0.85b	52.34 ± 0.76b
C5	1.6 ± 0.14f	53.69 ± 0.76c	51.2 ± 0.23c
C6	1.8 ± 0.77e	53.77 ± 0.91c	52.32 ± 0.48c
C	1.4 ± 0.11 g	52.5 ± 0.63d	50.23 ± 0.69f

**C1** = kitchen waste + fallen leaves (5–10 mm), **C2** = Kitchen waste + fallen leaves (1–3 mm), **C3** = Kitchen waste + saw dust (5–10 mm), **C4** = Kitchen waste + saw dust (1–3 mm), **C5** = Kitchen waste + fly ash (5–10 mm), **C6** = Kitchen waste + fly ash (1–3 mm). All the values are the mean of triplicate. *±* represents the Standard Deviation. The alphabet after numeric value represents the duncan multiple range test.

### Impact on organic matter content and C/N ratio of the compost

**Fig. 4:**
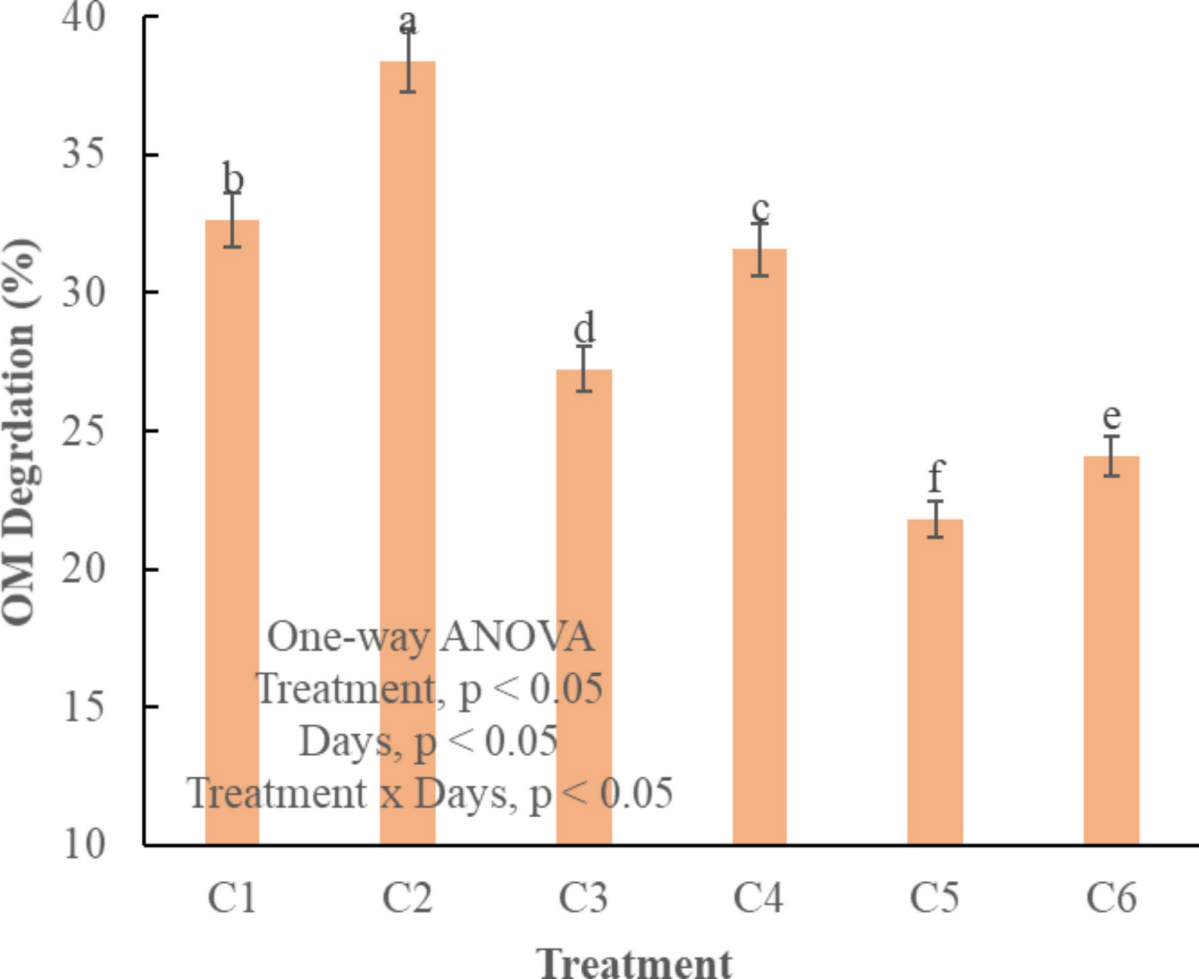
Organic matter degradation in different treatments of composting. C1 = kitchen waste + fallen leaves (5–10 mm), C2 = Kitchen waste + fallen leaves (1–3 mm), C3 = Kitchen waste + saw dust (5–10 mm), C4 = Kitchen waste + saw dust (1–3 mm), C5 = Kitchen waste + fly ash (5–10 mm), C6 = Kitchen waste + fly ash (1–3 mm). All the values are the mean of triplicate. Error bar represents the Standard Deviation. The alphabet above bars represents the duncan multiple range test.

Organic matter (OM) serves as a crucial indicator for assessing compost maturity, and there are significant differences among all treatments (*p* < 0.05) (Fig. 4). C2 exhibited the highest degradation rate and higher OM content at the end of composting process. Whereas, C5 and C6 displayed relatively slower degradation rates (Fig. 4). C5 and C6 are characterized as immature compost with an OM content of only 22%, falling below the desired range of 30–50% for compost maturity. The decline in OM content is due to its utilization by microbes as an energy source^40^ and food supply^35^. During this process the organic carbon is also lost through volatilization as CO_2_^14^. The absence of a bulking agent results in low degradation rates because of the absence of organic matter for microbial activity and growth^41^. Both fallen leaves and sawdust facilitated degradation of OM due to their large surface area for nurturing microbial activity^12^. Bulking agents with high adsorption capacity provide abundant nutrients for the microbial community^42^. Moreover, Fallen leaves also promoted enzymatic activities and decomposition of insoluble OM in the compost pile^43^.

**Fig. 5:**
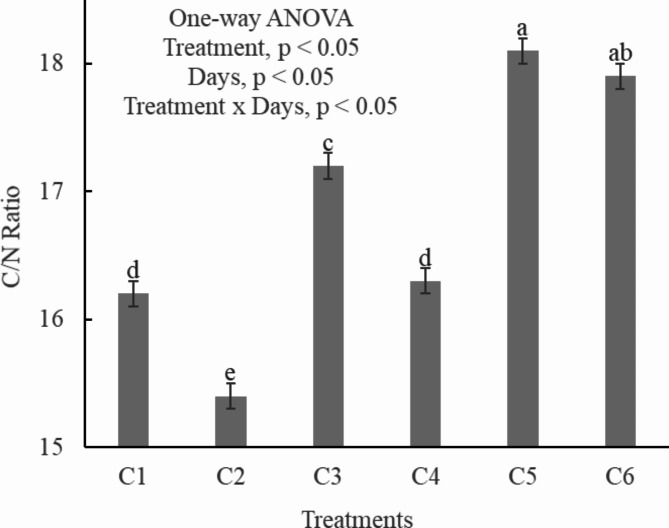
Carbon/nitrogen ration in different treatments of composting. C1 = kitchen waste + fallen leaves (5–10 mm), C2 = Kitchen waste + fallen leaves (1–3 mm), C3 = Kitchen waste + saw dust (5–10 mm), C4 = Kitchen waste + saw dust (1–3 mm), C5 = Kitchen waste + fly ash (5–10 mm), C6 = Kitchen waste + fly ash (1–3 mm). All the values are the mean of triplicate. Error bar represents the Standard Deviation. The alphabet above bars represents the duncan multiple range test.

Maintaining an optimum carbon-to-nitrogen (C/N) ratio is critical for effective composting and this is one of the potential indicator of compost maturity^44^. All the treatments showed statistically significant differences (*p* < 0.05) among each other for C/N ratio (Fig. 5). C5 exhibited a higher C/N ratio exceeding 18, this potentially leads to excessive mineralization or immobilization of nitrogen, which is detrimental for plant growth. In contrast, the C/N ratios for the other four treatments (C1, C2, C3 & C4) fell within the 14–18 range, this indicates the maturity of compost. Adjusting the waste-to-bulking agent ratio is necessary to optimize the carbon and nitrogen content of the compost^37^. An appropriate C/N ratio is vital for sustaining an active microbial population and accelerating the composting process^45^. A high C/N ratio can prolong the maturation period, while a lower ratio can result in rapid nitrogen losses through volatilization or runoff. The final compost’s C/N ratio should ideally range between 14 and 18, ensuring slow mineralization of nitrogen upon application to the soil^44^.

### NPK variation in compost

The NPK (nitrogen, phosphorus, and potassium) content is a critical factor in compost maturation and subsequently promote plant growth. In this study, all six treatments exhibited distinct nutritional values with significant differences (*p* < 0.05) as outlined in (Table 3). Treatment C2 demonstrated sufficient NPK levels, whereas C5 fell short of the targeted values. C5 and C6 displayed nearly identical NPK concentrations. These findings align with the results from the pot experiment, where stem height and leaf count increased in C2 and C3 treatments compared to the control. Elevated NPK ratios may induce nutrient immobility into the plant roots^36^, while lower ratios can lead to nutrient deficiencies in plants^43^.

**Table 3:** Maturity indices of final compost.

Treatments	Total phosphorous (%)	Total potassium (%)	Total Nitrogen (%)	Moisture Content (%)
C1	0.99 ± 0.01b	1.54 ± 0.04c	1.48 ± 0.21b	19.56 ± 1.41c
C2	1.14 ± 0.02a	1.61 ± 0.03a	1.46 ± 0.11c	18.25 ± 0.35 cd
C3	0.95 ± 0.11d	1.54 ± 0.09c	1.45 ± 0.23 cd	19.34 ± 1.41c
C4	0.97 ± 0.08c	1.59 ± 0.13b	1.49 ± 0.12a	18.25 ± 0.35d
C5	0.83 ± 0.04f	1.52 ± 0.21de	1.48 ± 0.03b	21.75 ± 0.35a
C6	0.87 ± 0.02e	1.53 ± 0.12d	1.48 ± 0.19b	20.05 ± 0.70b

**C1** = kitchen waste + fallen leaves (5–10 mm), **C2** = Kitchen waste + fallen leaves (1–3 mm), **C3** = Kitchen waste + saw dust (5–10 mm), **C4** = Kitchen waste + saw dust (1–3 mm), **C5** = Kitchen waste + fly ash (5–10 mm), **C6** = Kitchen waste + fly ash (1–3 mm). All the values are the mean of triplicate. *±* represents the Standard Deviation. The alphabet after numeric value represents the duncan multiple range test.

### Impact of compost on plant growth

**Fig. 6:**
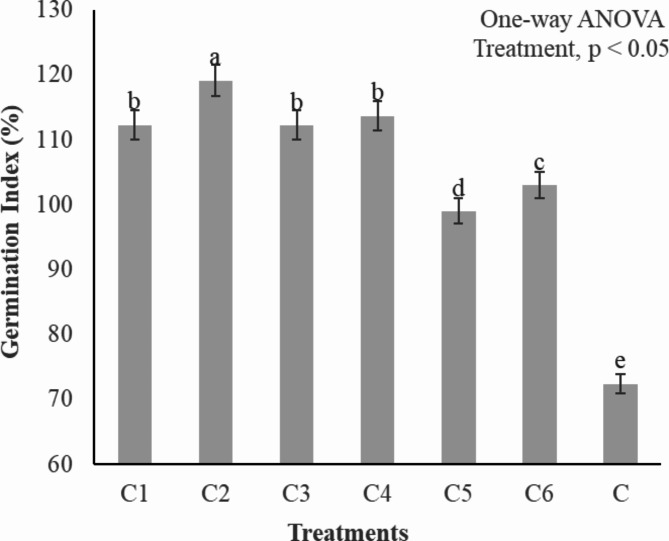
Variations in Germination Index (%) during different composting treatments. C1 = kitchen waste + fallen leaves (5–10 mm), C2 = Kitchen waste + fallen leaves (1–3 mm), C3 = Kitchen waste + saw dust (5–10 mm), C4 = Kitchen waste + saw dust (1–3 mm), C5 = Kitchen waste + fly ash (5–10 mm), C6 = Kitchen waste + fly ash (1–3 mm). All the values are the mean of triplicate. Error bar represents the Standard Deviation. The alphabet above bars represents the duncan multiple range test.

The determination of phytotoxicity is a critical factor in assessing compost maturity^46^. The present study evaluated whether the compost had reached maturity or still contained phytotoxic substances. Compost is considered mature when its Germination Index (GI) exceeds 80%^39^. The GI experiment involved six treatments, and their results showed significant differences (*p* < 0.05). germination index of all the treatments were above 80%, indicating the maturity of the compost (Fig. 6), (Table 4). The GIs for the C1, C2, C3 and C4 ranged from 119 − 112%, attributed to the combined properties of kitchen waste, fallen leaves and saw dust. Treatments C5 and C6 also showed the maturity signs but was of low then rest of the treatments. Bulking agents do contribute to the adsorption of toxic and volatile substances^34^. High GI can be attributed to the humic substances produced by bulking agents, which structurally stabilize root cell membranes^7^. Humic substances also enhance cell permeability for nutrient uptake^12^, resulting in improved plant growth^47^. Bulking agents, on the other hand, has a distinct mechanism with high water retention potential, leading to slow mineralization^48^ and reduced leachate production^46^. This, in turn, minimizes nutrient loss^40^ and promotes increased plant growth^42^.

**Table 4:** Changes in growth parameters in presence of different treatments.

Treatments	Plant height (cm)	No of Leaves	Fresh wt. of Shoot (g)	Fresh wt. of Root (g)
C1	15.55 ± 0.07b	20 ± 0.70a	4.7 ± 0.07b	0.99 ± 0.02b
C2	16.70 ± 0.14a	20 ± 1.41a	4.8 ± 0.07a	1.21 ± 0.21a
C3	15.45 ± 0.07b	18 ± 0.70c	4.6 ± 0.14c	0.98 ± 0.04b
C4	15.54 ± 0.01b	19 ± 0.70b	4.6 ± 0.14c	0.98 ± 0.01b
C5	14.35 ± 0.07c	17 ± 1.41d	4.3 ± 0.28e	0.91 ± 0.05d
C6	14.5 ± 0.14c	18 ± 0.70e	4.5 ± 0.07d	0.92 ± 0.07c
C	10.15 ± 0.21d	12 ± 0.70f	2.6 ± 0.14f	0.86 ± 0.01e

**C1** = kitchen waste + fallen leaves (5–10 mm), **C2** = Kitchen waste + fallen leaves (1–3 mm), **C3** = Kitchen waste + saw dust (5–10 mm), **C4** = Kitchen waste + saw dust (1–3 mm), **C5** = Kitchen waste + fly ash (5–10 mm), **C6** = Kitchen waste + fly ash (1–3 mm). All the values are the mean of triplicate. *±* represents the Standard Deviation. The alphabet after numeric value represents the duncan multiple range test.

### Impact of compost on soil properties

Addition of compost in soil increases the soil properties such as WHC, porosity and aggregate stability^49^. WHC of soil is the ability of soil to hold certain amount of water^5^. In this study, addition of composts increased the WHC of soil in all treatments as compared to control. Soil in T2 (amended with C2) have greater ability to hold water as compared to others. Water Holding capacity (2.8 g water/g dry sample) of soil was greater in T2 and lowest (1.6 g water/g dry sample) in T5 and in control was lower than other treatments. In this study, addition of composts increased the aggregate stability of soil in all treatments as compared to control. Aggregate stability of soil (54.4) was greater in T2 and lowest (51.2%) in T5 and in control was lower than other treatments. In this study, addition of composts increased the soil porosity in all treatments as compared to control. Porosity of soil (55.4%) was greater in T2 and lowest (53.6) in T5 and in control was lower than other treatments (Table 2) The statistically analysis showed significant difference in all treatments (*p* < 0.05). When soil aggregate stability is higher, its porosity is also higher^29^. Soil porosity is very important parameter and it is directly proportional to water and air in pores, required for plant^44^. Soil with increased porosity is beneficial for plant health. Aggregate stability of soil is its ability that have consequences for pore space suitable to regulate the flow of air and water, root growth and microbial activity. Al-Alawi et al.^50^ reported that the combination of GORE^®^ cover membrane and aerated static windrow is an efficient air-floor aeration system, this reduced the composting time to 30 days. The soil with high aggregate stability is good for plant growth as compared to soil with low aggregate stability^51^.

### Practical implications

From this study, it is recognized that all bulking agents (fallen leaves, saw dust, fly ash) can be used in kitchen waste composting to enhance composting process (Table 5). Yang et al.^12^, the effectively converted bean dregs into compost using wheat straw as bulking agent. Similarly, Rich et al.^25^, reported that saw dust is the best bulking agent compared to water hyacinth, garden prune and vegetable waste. Composting of lemon peel, cooked food waste and mixed vegetable waste can be completed by adding garden soil at house hold. The amendment with garden soil maintain the low thermophilic stage^51^. Bulking agents are easily available and studies have also reported the use of biochar as bulking agent. Materials that were successfully converted into biochar and used as bulking agents in composting process includes, wheat straw, rice straw, bamboo, corn cob, lignocellulose waste, wood chips, soft wood etc. Biochar based bulking agents improves the aeration, moisture retention, higher temperature retention, decrease in C/N value, reduced emission of N_2_O and CO_2_, increase in microbial development in compost^58^. In the same way, fly ash helps in enhancing the lignin decomposition^20^, reduces bio-availability of heavy metals^17^, neutralizes the acidic soil and facilitate microbial communities in compost^18^. By reducing size of kitchen waste, efficient degradation can be achieved. Composting of kitchen waste is a great way to recycle organic waste we generate at home. Composting of kitchen waste at household level is better option to manage it instead of landfilling. Composting process turn waste into something practical for our gardens. By adding compost in soil, health of soil improves and it also provide nutrients required by plants. Through composting, organic waste can be manage at home. Organic fertilizers are better than chemical fertilizers. Organic fertilizer does not contribute any hazardous compound to soil as compared to chemical fertilizer. It is better to make organic fertilizer from kitchen waste at home instead of buying expensive chemical fertilizers.

**Table 5:** Comparative table with previous studies.

Parameters	Values	Composting duration (days)	References
MC % C/N Ratio pH	56-63.9% < 12 8-8.6	21	Margaritis et al.^52^
C/N Ratio GI % pH	16–20 80–111% 7-8.5	28	Yang et al.^12^
pH C/N Ratio	6.7–7.1 12.08–18.4	30	Rich et al.^25^
MC % C/N Ratio GI %	45–70% 10–20 10–91%	30	Peng et al.^53^
**OM %** **C/N Ratio** **GI %** **pH**	**44.3–53.2%** **15.4–18.1** **99-119.1%** **7-8.5**	**32**	**This study.**
pH C/N Ratio GI %	8.6–9.6 11.1–15.6 82.7-106.7%	32	Li et al.^54^
MC % pH GI %	15.4–32.1% 7–9 80–130%	35	Ahmed et al.^7^
C/N Ratio GI % pH	9.1 127% 6.9	40	Zahrim et al.^55^
MC % pH P % K %	19.9 7.2 0.1 0.2	40	Ugak et al.^10^
C/N Ratio GI % pH K %	16–20 40.3-157.2% 8.5–8.9 1.2–1.5%	56	Zhou et al.^56^
pH EC mS/cm GI %	6.1–6.3 < 4 85%	60	Manu et al.^57^
pH C/N Ratio	7.6–7.8 7–29	70	Lalremruati and Devi^51^.

### Scalability and limitation of composting

Scalability of composting is explained as how effectively a system can manage the varying factors that are influencing the composting. This system must be sustainable, efficient, cost-effective and should produce quality compost^59^. Following are the factors that effects the scalability of compost, type of composting process, facility location, facility size, efficiency of technology in use, use of automation, type and quantity of waste material, collection system, local government regulation, community participation etc. Some of the mentioned factors are continuously changing and coping with them is a difficult task^60^. For industrial application new technology development is necessary for waste management. Al-Alawi et al.^50^ developed a new technology by combining GORE^®^ cover membrane and aerated static windrow for efficient air-floor aeration system. This new technology significantly reduced the composting time to 30 days. Similarly, encapsulated lifting system is a new technology to dispose of sewage slug water that was developed during composting process. This new technology consisted of 2 phases i.e. GORE^®^ cover membrane with aerated static windrow and a bottom up aeration system^61^.

### Environmental impact evaluation

The environmental impact evaluation of composting was not the objective of this study but it is multi-dimensional. On-site composting is considered the low-impact choice^62^. Quiros et al.^63^ reported the environmental superiority of home based compost over industrial based compost based on life cycle analysis. De Boni et al.^64^ stated that community scale composting projects produce environmental, social and economic benefits in small towns. Composting projects in Africa has reduced the greenhouse gases (GHG) by half as compared to the landfill gas combustion projects. Composting is eco-friendly but some activities (like segregation, packaging, transport etc.) uses more energy (about + 20%)^65^. In current economic situation the cost plays the crucial role in decision making, anaerobic digestion and composting collectively can increase the worth in terms of energy and finance^66^.

## Conclusion

Composting at home is a simple and useful method to treat kitchen waste. This research was conducted to examine the effect of kitchen waste size and three different bulking agents on degradation of kitchen waste through aerobic composting at household level, and impact of final compost on plant growth and soil properties. Number of materials are successfully converted into biochar subsequently used as bulking agents in composting process. These includes, wheat straw, rice straw, bamboo, corn cob, lignocellulose waste, wood chips, soft wood etc. Biomass fly ash and coal fly ash has the potential to neutralize the acidic soil and reduce the number of exchangeable hydrogen ions. It was observed that all treatments gave matured and phytotoxic free compost on day 32, but the treatment (C2) in which kitchen waste size was small and fallen leaves were used as bulking agent provided better results than other treatments due to its maximum positive impacts on composting process. C2 also has more positive impacts on plant growth and soil properties as compared to other treatments. This study is useful in optimizing kitchen waste management by reducing kitchen waste size and using fallen leaves (easily provided) as bulking agent at household level to convert kitchen waste into valuable product in a small period of time (32 days) that can be used as soil fertilizer. Number of varying factors effects the scalability of compost, type of process, facility location, facility size, technology efficiency, use of automation, type and quantity of waste material, collection system, local government regulation, community participation etc. The environmental impact evaluation of composting is multi-dimensional, community scale composting projects produce environmental, social and economic benefits in small towns. Composting has potential to reduce greenhouse gases (GHG) by half as compared to the landfill gas combustion projects.

## Data Availability

All data generated or analyzed during the study are included in this article.
